# Analyzing network diversity of cell–cell interactions in COVID-19 using single-cell transcriptomics

**DOI:** 10.3389/fgene.2022.948508

**Published:** 2022-08-29

**Authors:** Xinyi Wang, Axel A. Almet, Qing Nie

**Affiliations:** ^1^ Department of Mathematics, University of California, Irvine, Irvine, CA, United States; ^2^ The NSF-Simons Center for Multiscale Cell Fate Research, University of California, Irvine, Irvine, CA, United States; ^3^ Department of Developmental and Cell Biology, University of California, Irvine, Irvine, CA, United States

**Keywords:** network analysis, single-cell, cell–cell interactions, diversity, COVID-19

## Abstract

Cell–cell interactions (CCI) play significant roles in manipulating biological functions of cells. Analyzing the differences in CCI between healthy and diseased conditions of a biological system yields greater insight than analyzing either conditions alone. There has been a recent and rapid growth of methods to infer CCI from single-cell RNA-sequencing (scRNA-seq), revealing complex CCI networks at a previously inaccessible scale. However, the majority of current CCI analyses from scRNA-seq data focus on direct comparisons between individual CCI networks of individual samples from patients, rather than “group-level” comparisons between sample groups of patients comprising different conditions. To illustrate new biological features among different disease statuses, we investigated the diversity of key network features on groups of CCI networks, as defined by different disease statuses. We considered three levels of network features: node level, as defined by cell type; node-to-node level; and network level. By applying these analysis to a large-scale single-cell RNA-sequencing dataset of coronavirus disease 2019 (COVID-19), we observe biologically meaningful patterns aligned with the progression and subsequent convalescence of COVID-19.

## 1 Introduction

Cell–cell interactions (CCI) manipulate multiple biological processes, including organismal development, homeostasis, and immune responses (Armingol et al., 2021). When cells do not interact properly, disease occurs (Armingol et al., 2021). The recent advances in single-cell RNA-sequencing (scRNA-seq) offer great opportunities to decipher CCI through coordinated gene expression of ligand–receptor pairs. Using a diverse range of strategies, many tools have been designed to infer CCI from scRNA-seq (Almet et al., 2021; Armingol et al., 2021). These computational tools can be grouped into four categories: differential combination–based, network-based, permutation-based, and array-based (Armingol et al., 2021). The majority of CCI inference methods are designed to analyze CCI for a single condition. As characterizing differences in CCI activity across multiple conditions—for example, between healthy and diseased tissue—helps elucidate the diverse mechanisms of CCI-mediated responses to disease, more so than analyzing condition-specific CCI networks in isolation, recent efforts have focused on developing methodologies to systematically study the differences in CCI between multiple conditions (Xiong et al., 2019; Gibbs et al., 2021; Wang et al., 2022). However, the majority of previous CCI analyses that analyze differences between conditions tend to analyze CCI networks inferred from a single scRNA-seq sample from each condition or analyze CCI networks constructed by aggregating several samples. These analyses result in deterministic characterizations of CCI and fail to take into account the underlying variability across the groups of samples that constitute each biological condition.

As single-cell omics field is expanding, data are becoming more readily accessible. Advances in sequencing technologies have enabled more sample replicates to be obtained at cheaper prices and a greater number of cells that can be sequenced per replicate (Svensson et al., 2018). While including multiple samples can obscure biological variation *via* batch effects (Luecken et al., 2022), having multiple samples per condition enables one to characterize the variability of key aspects such as CCI at the “group level”, where multiple samples comprise a single condition. Being able to account for variability across samples, such as those sampled from human patients, is particularly important for diseases where host response is associated with severity of disease progression, such as COVID-19 (Ong et al., 2020). Indeed, there are a number of scRNA-seq studies of COVID-19 where multiple samples have been obtained for both healthy and diseased status (Liao et al., 2020; Zhang et al., 2020; Melms et al., 2021; Ren et al., 2021; Stephenson et al., 2021; Kuchroo et al., 2022). In particular, Ren et al. (2021) produced scRNA-seq data containing 1.5 million cells from 196 patients across five COVID-19 conditions. As more large-cohort scRNA-seq studies become available, it is important to develop methodologies that account for and analyze the sample-to-sample variability within and across biological conditions.

Thus, in this article, we propose a study on diversities of three classes of features of CCI networks within groups of different conditions. More specifically, since a CCI network can be represented as a directed weighted graph, where the nodes correspond to cell states and edge weights correspond to interaction strengths, we can extract CCI features at three levels: single node, node-to-node, and whole graph. We apply these methods to a large single-cell transcriptomic COVID-19 dataset comprising peripheral blood mononuclear cells (PBMC) sampled from 181 patients and analyze CCI diversity patterns under different COVID-19 conditions. By analyzing each specific CCI feature and comparing its diversity across different sample groups comprising biological conditions, we are able to observe consistent and biologically meaningful patterns aligned with the progression and subsequent convalescence of COVID-19.

## 2 Materials and methods

In this section, we have described the dataset we used, the CCI analysis from CellChat, and the methods that we implemented to analyze CCI diversity.

### 2.1 Data preparation

The scRNA-seq data of COVID-19 progression were downloaded from the NCBI GEO database, accession number GSE158055. The original scRNA-seq data consist of samples taken from PMBCs, bronchoalveolar lavage fluid (BALF), and sputum. To ensure consistency in biological sample type as well as cell type, we only retained PBMCs. The considered data consist of 181 COVID-19 patients and controls, including 25 healthy controls, 18 patients with moderate symptoms, 43 patients with severe symptoms, 57 patients in the convalescent stage from moderate symptoms, and 38 patients in the convalescent stage with severe symptoms. All single-cell preprocessing and analysis were performed using Seurat (Hao et al., 2021). Quality control was performed according to the original study (Ren et al., 2021). Specifically, cells with fewer than 1,000 unique molecular identifier (UMI) counts and 500 detected genes were removed, as well as cells with more than 10% of gene counts arising from mitochondrial genes. To remove potential doublets, we also filtered out cells that contained more than 25,000 UMI counts and 5,000 detected genes. In addition, we used Scrublet (Wolock et al., 2019) to identify doublets. The expected doublet rate was set to 0.08, and cells predicted to be doublets were removed. Prior to dimensionality reduction, we determined the 1,500 most highly variable genes. Gene expression counts were normalized to 10,000 counts per cell, log-transformed, and then scaled. We used the original clustering metadata to label B cells, T cells, and myeloid cells (Ren et al., 2021).

### 2.2 CCI analysis by CellChat

To infer significant CCI from the patient-specific scRNA-seq data, we used CellChat (Jin et al., 2021). CellChat infers significant CCI activity from scRNA-seq data between identified cell groups, such as cell states or cell types. CellChat calculates an interaction score based on mass action kinetics that reflects the likelihood of CCI by integrating gene expression with prior knowledge of the interactions between signaling ligands, receptors, and their cofactors (Jin et al., 2021). CCI is identified at two levels: at the level of single ligand–receptor pairs and at the aggregate level of signaling pathways that consist of multiple ligand–receptor interactions. The calculated interaction strength between cell group *i* and cell group *j* between a particular ligand–receptor pair is between 0 and 1. Assuming *N* cell types have been identified in the data, the result of the inference is a CCI network where each node *i* ∈ {1, 2, … , *N*} represents a cell type and interactions are encoded by the weighted adjacency matrix, *A*. Therefore, each entry, *A*_*ij*_, is a directed weighted edge that represents the strength of interaction from the sender cell type *i* to the receiver cell type *j*.

CellChat provides functionality to quantify and compare relevant biological features of a single CCI network and between pairs of networks. For this study, we used CellChat to calculate the information flow, network centrality, functional similarity, and structural similarity between pairs of CCI networks. Here, we briefly describe each method.

The information flow of a network in CellChat is computed by the following formula:

$$I_{f}=-{\left[\log\left(\overset{N}{\underset{i,j=1}{\sum }}A_{ij}\right)\right]}^{-1},$$ (1)

where the log is taken to amplify small differences in interaction scores. The information flow, *I*_*f*_, quantifies the total strength of interactions in a network.

CellChat also allows for a network centrality analysis on the CCI networks. In each directed and weighted CCI network, the in-degree centrality, out-degree centrality, flow betweenness centrality, and information centrality measure can be calculated to identify important receivers, senders, mediators, and influencers in the network (Jin et al., 2021).

The functional similarity between two networks is calculated using the Jaccard similarity (Jin et al., 2021):

$$S=\frac{\left|E\left(G\right)\cap E\left(G^{\prime }\right)\right|}{\left|E\left(G\right)\cup E\left(G^{\prime }\right)\right|},$$ (2)

where *G* and *G*′ are two signaling networks and *E*(*G*) and *E* (*G*′) are the edge sets describing the set of interactions in signaling networks *G* and *G*′, respectively. It quantifies the similarity of major sender and receivers of each network. The structural similarity is used to measure a topological similarity between the structures of two networks. It is based on a previous measure on topological similarity (Schieber et al., 2017).

The structural similarity, *S*, between two graphs, *G* and *G*′, with *N* and *M* cell types, respectively, is defined with respect to the structural dissimilarity, *S* (*G*, *G*′) = 1 − *D* (*G*, *G*′), which is defined as follows:

$$\begin{array}{ll} D\left(G,G^{\prime }\right) & =w_{1}\sqrt{\text{JSD}\left(u_{G},u_{G^{\prime }}\right)/\log2}+w_{2}|\sqrt{\text{NND}\left(G\right)}-\sqrt{\text{NND}\left(G^{\prime }\right)}|\\ & \;+\frac{w_{3}}{2}\left(\sqrt{\text{JSD}\left(P_{\alpha G},P_{\alpha G^{\prime }}\right)/\log2}+\sqrt{\text{JSD}\left(P_{\alpha G^{c}},P_{\alpha G^{c^{\prime }}}\right)/\log2}\right), \end{array}$$ (3)

where *w*_1_, *w*_2_, and *w*_3_ are the weights such that *w*_1_ + *w*_2_ + *w*_3_ = 1. The Jensen–Shannon divergence, JSD, is defined across *N* probability distributions, *P*_1_, … , *P*_*N*_, as follows:

$$\text{JSD}\left(P_{1},\ldots ,P_{N}\right)=\frac{1}{N}\overset{N}{\underset{i,j}{\sum }}p_{i}\left(j\right)\log\left(\frac{p_{i}\left(j\right)}{u_{j}}\right),\;u_{j}=\frac{1}{N}\overset{N}{\underset{i=1}{\sum }}p_{i}\left(j\right),.$$ (4)

where *u*_*j*_ is the average across the *N* probability distributions.

In Eq. 3, JSD (*u*_*G*_, *u*_*G*’_) is the Jensen–Shannon divergence between the averages of the cell type distance distributions of signaling graphs; JSD (*P*_*αG*_, *P*_*αG*′_) is the Jensen–Shannon divergence between the *α*-centrality (Katz centrality) values of *G* and *G*′; and $\text{JSD}\left(P_{\alpha G^{c}},P_{\alpha G^{c^{\prime }}}\right)$ is the Jensen–Shannon divergence between the *α*-centrality values of the graph complements of *G* and *G*′ and *G* ^*c*^ and *G* ^*c*^’, respectively, where for a graph, *G*, with *N* vertices, the graph complement, *G* ^*c*^, is defined by the same vertex set, *V* (*G* ^*c*^) = *V*(*G*), but the edge set is constructed by *E* (*G* ^*c*^) = *E* (*K*_*N*_)\*E*(*G*), where the complete graph, *K*_*N*_, is the graph constructed by connecting all distinct pairs of vertices.

Finally, NND is the network node dispersion (Schieber et al., 2017) defined over the distance distributions of the *N* cell types, *P*_*i*_ = {*p*_*i*_(*j*)}, encoded by *G*:

$$\text{NND}\left(G\right)=\frac{\text{JSD}\left(P_{1},\ldots ,P_{N}\right)}{\log\left(d+1\right)},$$ (5)

where *d* is the diameter of the network.

Next, to learn a shared space for pathway classification, manifold learning of different signaling CCI networks is performed through the following steps. First, a shared nearest-neighbor similarity network, *Gs*, of CCI networks is constructed by calculating the *k*-nearest signaling pathways of each pathway with respect to the functional or structural similarity matrix, *S*. The weights of the shared nearest-neighbor network are calculated as the fraction of shared nearest signaling pathways between a given pathway and its neighbors. Next, the similarity matrix, *S*, is smoothed by calculating *Gs* × *S*. Finally, uniform manifold approximation and projection (UMAP) is performed on the smoothed similarity matrix.

To adapt these methods and analyze the differences across the five considered COVID-19 conditions, we first combined scRNA-seq data of all patients in each condition group as a single aggregated sample. We interpret the resulting CCI network as describing the CCI within an average patient in each group.

### 2.3 Measures of graph features

In this section, we devise and implement measures of single node and between-node features of a CCI network to compare the diversity of these features across all COVID-19 conditions. Rather than using the methods described in Section 2.2 for aggregated scRNA-seq data, we used CellChat to obtain the CCI network for each individual patient, and the following analysis was performed based on these 181 data points.

#### 2.3.1 Single node features in the CCI network

For each sender or receiver cell type, we considered three measures of diversity:

1) Node degree. The weighted out-degree of a sender cell type is the total amount of interactions of the sender cell type. For receiver cell types, we considered the weighted in-degree.
2) Node diversity. The diversity of a sender cell type is the number of different receiver cell types with which it interacts. Conversely, the diversity of a receiver cell type is the number of different receivers with which it interacts.
3) Node entropy. The entropy of a sender (receiver) cell type measures how balanced or uniform the distribution of its outgoing (incoming) interactions are (Shannon, 1948). This measure of how balanced or uniform the relevant outgoing (incoming) interactions are depends on the relative interaction strengths between one sender cell type and relevant different receiver (sender) cell types. For example, if the outgoing interaction strengths of one sender are spread evenly across all receivers, then the entropy will be low. On the contrary, if the outgoing interactions from a sender are highly concentrated toward one receiver, then the entropy will be high. As entropy depends on the relative strengths, rather than absolute strengths, we first normalized the interaction strengths of each sender cell type *i*:

$${\tilde{A}}_{ij}=\frac{A_{ij}}{\sum_{j=1}^{n}A_{ij}}.$$ (6)

For the case when a sender cell does not interact with any receivers, we defined ${\tilde{A}}_{ij}=0$ . The (outgoing) entropy of a sender cell type, *i*, is then calculated using Shannon entropy (Shannon, 1948):

$$H_{\mathrm{sender}}\left(i\right)=-\overset{n}{\underset{j=1}{\sum }}{\tilde{A}}_{ij}\log\left({\tilde{A}}_{ij}\right),$$ (7)

The (incoming) entropy of receiver cell types is calculated similarly:

$$H_{\mathrm{receiver}}\left(i\right)=-\overset{n}{\underset{j=1}{\sum }}{\tilde{A}}_{ji}\log\left({\tilde{A}}_{ji}\right),$$ (8)

We noted that the entropy is always positive. For cases where a sender (receiver) has no outgoing (incoming) interactions, we treat its entropy as zero.

#### 2.3.2 Proximity measures between CCI node pairs

In this section, we have described three measures for node-to-node or cell-type-to-cell-type features.

##### 2.3.2.1 Information flow proximity matrix

Cell signaling can be interpreted as the flow of biochemical information between cells (Azeloglu and Iyengar, 2015). Thus, based on several assumptions, we can consider the CCI network as an information exchange network. As the communication score is based on the level of relevant gene expression and a higher level of relevant ligand and receptor expression reflects more significant interaction, a higher interaction, in turn, implies that more information from sender cell type *i* flows to receiver cell type *j*. Furthermore, we assumed that if the interaction strength between cell type *i* and type *j* is 1, then the quantity of information flow reaches its maximum. For a network described by the directed, weighted adjacency matrix, *A*, each interaction strength, *A*_*ij*_, can be viewed as a proportion in the maximal amount of information that cell type *i* can send to cell type *j*. Second, we assumed that the information flow does not only happen within a direct neighborhood of cells but may also flow through intermediate cell types. For example, some information from cell type *i* to cell type *k* can first flow to cell type *j* and then arrive at cell type *k*. Therefore, it is natural to assume that the information flow can be aggregated among all paths from cell *i* to *j*.

Now, we may consider the information flow from cell type *i* to *j* along all possible (directed) paths within the network. Such a propagation of information can take *n* steps with *n* − 1 intermediate nodes; in general, ${\left(A^{n}\right)}_{ij}$ measures the information flow from node *i* to node *j* in *n* steps. Note that we may sum the information flow along all paths from *i* to *j*, since flows along each path are independent of each other. Under this framework, to measure the total amount of information exchange from any cell type *i* to cell type *j* across the network, we can define the information flow proximity matrix, *S*, as follows:

$$S=\overset{\infty }{\underset{n=1}{\sum }}A^{n}={\left(I-A\right)}^{-1}-I,$$ (9)

where I is the identity matrix. Each entry, S_ij_, measures the total information flow from node i to j. It measures how much information is lost to node *j* if node *i* is removed from the network. Thus, we interpret the information flow as a network-level measure of importance of cell type *i* to *j*, as opposed to individual-level importance, which is characterized by the CCI strength.

##### 2.3.2.2 Modified Canberra proximity matrix

It is fair to assume that the interactions of a sender with different receivers are mutually independent. Thus, a more reasonable way to compare the differences in outgoing interactions between two senders with each individual receiver is to consider the relative difference between each interaction, instead of the absolute differences. We measured the relative difference of two senders or receivers using the modified Canberra distance.

For two sender cell types, we can represent all outgoing interactions by interaction vectors, **u** = (*A*_*i*1_, … , *A*_*in*_), **v** = (*A*_*j*1_, … , *A*_*jn*_). The original Canberra distance is defined as the sum of all relative pairwise distances between two interaction vectors (Lance and Williams, 1966):

$$d_{\mathrm{Can}}\left(u,v\right)=\underset{k}{\sum }\frac{\left|A_{ik}-A_{jk}\right|}{\left|A_{ik}|+|A_{jk}\right|},$$ (10)

where *k* is summed over all entries of the interaction vectors such that *A*_*ik*_ ≠ 0 and *A*_*jk*_ ≠ 0. However, in practice, the interaction vectors are often sparse. Therefore, when both *u* and *v* are zero for the same entry, that is, *A*_*ik*_ = *A*_*jk*_ = 0 for some *k*, the receiver *k* does not provide information about sender similarity. Therefore, we choose to use the modified Canberra distance. The modified Canberra distance between two sender cell types is defined as follows:

$$d_{\mathrm{ModCan}}\left(u,v\right)=\frac{1}{m}d_{\mathrm{Can}}\left(u,v\right),$$ (11)

where 0 ≤ *m* ≤ *n* is the number of terms in the summation of Eq. 10. Canberra distance is sensitive to small changes when both entries are near zero (Kaur, 2014), and the modified Canberra distance inherits this characteristic straightforwardly. A small modified Canberra distance between two sender cell types can be interpreted as the two senders having similar outgoing interaction strengths across all other cell types.

We then define the modified Canberra similarity between **u** and **v** as 1 − *d*_*ModCan*_(**u**, **v**). By considering all pairwise modified Canberra similarities between sender cells, we can construct the modified Canberra sender proximity matrix, *D*_*ModCan*_(*s*), where the entries ${\left(D_{\mathrm{ModCan}}\left(s\right)\right)}_{ij}$ represent the modified Canberra similarities between sender cell type *i* and *j*. We defined modified Canberra receiver proximity matrix, *D*_*ModCan*_(*r*), similarly.

##### 2.3.2.3 Weighted cosine proximity matrix

As signaling pathways consist of multiple interacting ligand–receptor pairs, it is natural to expect that not all ligands, or receptors, contribute equally to CCI activity. To characterize the similarity of outgoing (incoming) interactions between two senders (receivers), we may ask if both senders (receivers) contain a similar composition of pathway-specific ligands (receptors) but only differs in the level of expression. To quantify this similarity, we used the interaction strength between a sender and all receivers, as inferred from CellChat. For sender cell types, *i* and *j*, we represent the outgoing interactions by the vectors, **u** = (*A*_*i*1_, … , *A*_*in*_) and **v** = (*A*_*j*1_, … , *A*_*jn*_), respectively. Similarly, if *i* and *j* were both receivers, the incoming interactions can be represented by the two vectors, **u** = (*A*_1*i*_, … , *A*_*ni*_) and **v** = (*A*_1*j*_, … , *A*_*nj*_), respectively. If **u** and **v** lie in the same direction, meaning that the outgoing interactions from sender *i* and *j* to all receivers are similar, then it is reasonable to believe that sender *i* and *j* express the same composition of ligands. To characterize the similarity of directions between **u**, **v**, we used the cosine similarity. However, in practice, the interaction vectors **u** and **v** are often sparse. Therefore, when both **u** and **v** are zero for the same entry, that is, *A*_*ik*_ = *A*_*jk*_ = 0 for some *k*, the receiver *k* does not provide information about sender similarity. This observation motivates the use of the weighted cosine similarity:

$${Cos}_{\mathrm{weighted}}\left(u,v\right)=\frac{m}{n}\cdot \frac{u\cdot v}{\left\|u\|\|v\right\|},$$ (12)

where 0 ≤ *m* ≤ *n* is the total number of non-zero entries. Compared with the traditional unweighted cosine similarity, the weighted cosine similarity characterizes the certainty of the similarity measure between two interaction vectors, providing a more reliable measure of node-to-node similarity.

The weighted cosine sender proximity matrix consists of pairwise (sender cell type to sender cell type) weighted cosine similarities; that is, the *i*-*j*th entry represents the weighted cosine similarity between sender cell type *i* and *j*. We defined the weighted cosine receiver proximity matrix in a similar manner.

#### 2.3.3 Variability measures of CCI network groups

In this section, we introduced different ways to measure distance between CCI networks. Using these distance measures, we calculated the variability of networks within each group of COVID-19 conditions correspondingly.

##### 2.3.3.1 Root euclidean distance between proximity matrices

Having defined node-to-node similarity measures, it is valuable to have a measure of network-to-network similarity. We used the root Euclidean distance to measure the distance between node-to-node proximity matrices *S* ^(1)^ and *S* ^(2)^ (Koutra et al., 2013):

$$d_{r}\left(S^{\left(1\right)},S^{\left(2\right)}\right)=\sqrt{\overset{n}{\underset{i,j=1}{\sum }}{\left(\sqrt{S_{ij}^{\left(1\right)}}-\sqrt{S_{ij}^{\left(2\right)}}\right)}^{2}}.$$ (13)

Compared to the traditional Euclidean distance, *d*_*r*_ enlarges the difference when the entries are close to zero. This property of the root Euclidean distance allows one to discern differences between networks even when the interactions are small.

Based on the root Euclidean distance, we studied the variability of CCI networks under different disease conditions. For a condition-specific group of patients with CCI networks *A* ^(1)^, *A* ^(2)^, …, *A* ^(*k*)^, we used the following definition as the measure of within-group variability:

$${\text{Var}}_{r}\left(A^{\left(1\right)},A^{\left(2\right)},\ldots ,A^{\left(k\right)}\right)=\frac{1}{k-1}\underset{i=1,\ldots ,k}{\min}\underset{j\neq i}{\sum }d_{r}\left(S^{\left(i\right)},S^{\left(j\right)}\right),$$ (14)

where *S* ^(*i*)^ can be either the information flow, modified Canberra, or weighted cosine proximity matrix of *A* ^(*i*)^.

##### 2.3.3.2 Modified Canberra distance between networks

The modified Canberra distance between two CCI networks represented by adjacency matrices *A* and *B* across the same cell types is defined as follows:

$$d_{\mathrm{ModCan}}\left(A,B\right)=\frac{1}{m}\underset{i,j}{\sum }\frac{\left|A_{ij}-B_{ij}\right|}{\left|A_{ij}|+|B_{ij}\right|},$$ (15)

where the summation is over *i* and *j* such that *A*_*ij*_ ≠ 0 or *B*_*ij*_ ≠ 0, and *m* is the number of non-zero entries. Similar to the previous definition of modified Canberra distance for sender cell types (Equation (11)), a small modified Canberra distance implies that two CCI networks are similar with respect to their interaction strengths.

Based on the modified Canberra distance between networks, we studied the variability of CCI networks under different disease conditions. For a condition-specific group of patients with CCI networks *A* ^(1)^, *A* ^(2)^, …, *A* ^(*k*)^, where *A* ^(*i*)^ is the adjacency matrix of *ith* network, we used the following definition as the measure of within-group variability:

$${\text{Var}}_{\mathrm{ModCan}}\left(A^{\left(1\right)},A^{\left(2\right)},\ldots ,A^{\left(k\right)}\right)=\frac{1}{k-1}\underset{i=1,\ldots ,k}{\min}\underset{j\neq i}{\sum }d_{\mathrm{ModCan}}\left(A^{\left(i\right)},A^{\text{(}j\text{)}}\right).$$ (16)

## 3 Results

We applied the methods described to analyze the diversity of CCIs across different COVID-19 conditions. We first applied the analysis from CellChat to analyze difference of conditions comprising aggregated samples. We then analyzed the within-condition heterogeneity of CCI networks using the three categories of our developed measures: single node level, node-to-node level, and whole graph level.

### 3.1 Analysis of CCI across different COVID-19 conditions

To study CCI differences across COVID-19 patients under different disease conditions, such as healthy, moderate disease, severe disease, convalescent from moderate disease, and convalescent from severe disease, we used CellChat and studied the interactions among all 3 cell types: myeloid, B cells, and T cells.

**FIGURE 1:**
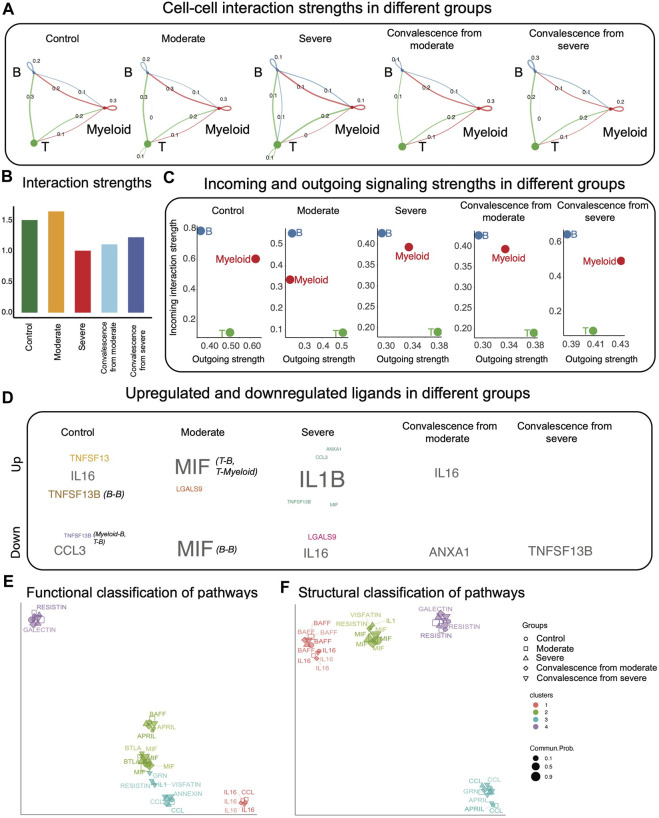
CCI analysis using CellChat. **(A)** CCI strengths. Each edge represents an interaction; the color of the edge matches with the sending cell type. **(B)** Total interaction strength in different groups. Control and moderate groups have higher total interaction strength. **(C)** Total incoming and outgoing signaling strengths in different groups. B cells interact stronger as receivers than as senders, while T cells interact stronger as senders than as receivers. **(D)** Upregulated and downregulated ligands in different groups. **(E)** Using UMAP to project signaling pathways on a two-dimensional manifold according to their functional similarity. Each dot represents the interaction network of one signaling pathway. Different colors represent different groups of signaling pathways. **(F)** Using UMAP to project signaling pathways on a two-dimensional manifold according to their structural similarity. Each dot represents the interaction network of one signaling pathway. Different colors represent different groups of signaling pathways.

CCI between any two cell types consist of interactions *via* different signaling pathways. First, we compared the CCIs of each cell pair in five groups (Figure 1A). We observed that autocrine signaling of T cells only exists in moderate and severe groups. Myeloid autocrine signaling decreased in severe samples, compared to healthy controls and moderate samples. In samples in the convalescence stage from severe disease, myeloid autocrine was recovered compared to the severe group. Compared to healthy controls and moderate patients, signaling from T cells to B cells was decreased in the rest of the condition groups. Interestingly, interaction strengths from T cells to myeloid cells were consistent over all the five groups. We then examined the differences in total interaction strengths in the five groups (Figure 1B) and found that the interaction strengths were higher in the control and moderate groups and the lowest in the severe group. We also compared the incoming and outgoing interaction strengths of different cell types (Figure 1C). The total interaction strength of B cells as a receiver is always stronger than as a sender, while the total interaction strength of T cells as a sender is always stronger than as a receiver. Myeloid cells interact similarly in strengths as senders and receivers.

Having obtained a general knowledge on CCI strengths across five conditions, we then investigated upregulated and downregulated signaling in each group compared with the remaining four groups (Figure 1D). CellChat predicted significant upregulation of three ligand–receptor pairs in control patients, consisting of the ligands TNFSF13, IL16, and TNFSF13B, which were sent by B cells and received by B cells; significant downregulation of ligand–receptor in control patients, consisting of the ligands CCL3 and TNFSF13B, which are sent from myeloid cells to B cells and from T cells to B cells. We also observed five upregulated ligand–receptor pairs in severe patients, with the corresponding ligands being IL1B, ANXA1, CCL3, TNFSF13B, and MIF, and two dowregulated ligand–receptor pairs, where the ligands are IL16 and LGALS9. Interestingly, there were no upregulated ligand–receptor pairs in the severe convalescence group.

Next, we examined functional and structural similarities of different signaling pathways in five disease conditions (Figures 1E,F). We observed that most pathways were grouped together under both similarity measures even if they were of different disease conditions.

To test robustness of CCI output and the conclusions from CellChat, we downsampled the original data to 80% of each original condition groups. We found that the CCI networks were robust to downsampling (Figure 1A, Supplementary Figure S1A), as had been found previously in Jin et al. (2021). The majority of conclusions from CellChat hold under downsampling. However, the upregulated and downregulated ligands change (Figure 1D, Supplementary Figure S1C). Specifically, the upregulated genes all disappear in the downsampled version, and downregulated ligands in moderate and convalescence from severe group are different.

### 3.2 Analysis of the MIF signaling pathway

**FIGURE 2:**
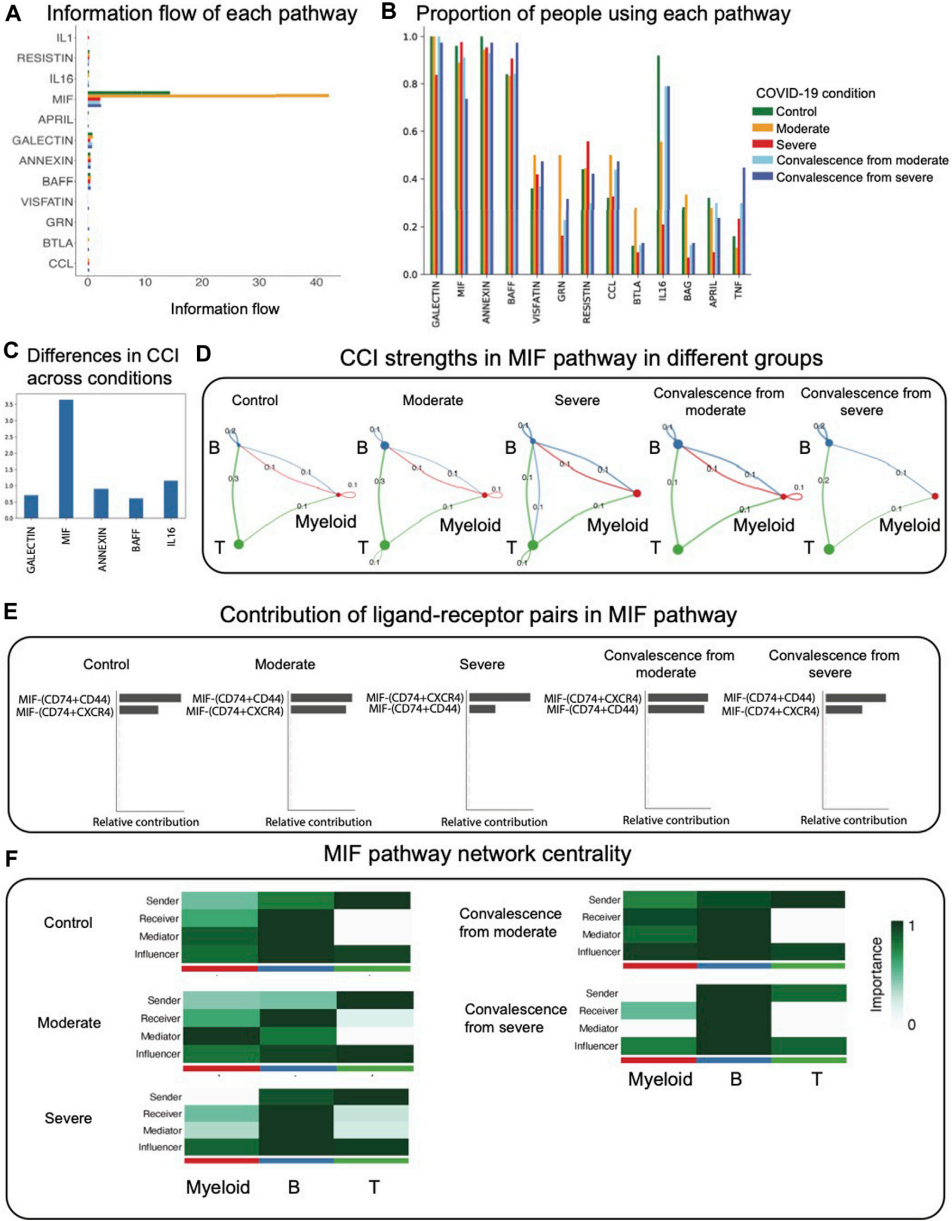
CCI analysis using CellChat focused on the MIF pathway. **(A)** Information flow (total interaction strengths) of each pathway. The MIF pathway contains the most amount of information. **(B)** Proportion of people using each pathway in different groups. The majority of CCI occurs through GALECTIN, MIF, ANNEXIN, and BAFF. **(C)** Average pairwise Canberra distance of CCI across conditions in the popular pathways. **(D)**. CCI strengths in the MIF pathway. Each edge represents an interaction; the color of the edge matches with the sending cell type. **(E)** Relative contribution of ligand–receptor pairs in the MIF pathway. **(F)** Network centrality scores of the MIF pathway. In severe and convalescence from severe groups, myeloid cells lose its function as a mediator.

The log-scaled information flow calculated by CellChat is shown in Figure 2A. We observed that the MIF pathway contains the highest total interactions over all signaling pathways, while other pathways contain little amount of interactions. We then calculated the percentage of patients in different groups using each pathway (Figure 2B). Out of all pathways, we found that the GALECTIN, MIF, ANNEXIN, BAFF, and IL16 pathways were present in more than 60% of patients, while other inferred pathways were present in fewer than 60% of patients in each condition. To find the most differential pathway across conditions among these popular pathways, we calculated pairwise Canberra distance of CCI in these pathways between each condition and took average among all pairs. We observed that the MIF pathway is also the most differential pathway across conditions (Figure 2C). Furthermore, as recent studies have shown that there is a strong correlation between the MIF serum level and COVID-19 severity, suggesting that the MIF serum level may be a useful predictor of COVID-19 disease severity (Aksakal et al., 2021; Bleilevens et al., 2021; Dheir et al., 2021), we focused our analysis on CCI diversities in the MIF pathway for the remainder of this study.

We observed that in the MIF pathway, autocrine signaling of T cells only exists in two diseased groups, which indicates that most T cells’ autocrine signaling in total CCI comes from the MIF pathway (Figure 2D). We found that unlike the rest of the groups, myeloid cells exhibit no autocrine signaling in severe and convalescence from severe groups. We also observed an interaction from B cells to T cells occurring in the severe group, which does not exist in the rest of the groups. We found that in the MIF pathway, the interaction from T cells to B cells was the highest interaction, except for convalescence from severe group in which it is the second highest. This indicates that T to B signaling functions as the major interaction in the MIF pathway. The most active ligand–receptor pairs in the MIF pathway are then compared across conditions (Figure 2E). In the control, moderate, and convalescence from severe conditions, the most active multi-receptor unit is the heteromeric complex containing CD74 and CD44. However, in the severe and convalescence from moderate conditions, the most active multi-receptor unit is the heteromeric complex containing CD74 and CXCR4, indicating differences in the types of MIF-specific interactions across COVID-19 conditions.

Next, applying the network centrality analysis of the MIF signaling network shows that in severe and convalescence from severe groups, myeloid cells lose their function as a mediator (Figure 2F). This indicates that in those two groups, the myeloid cells have a diminished role as a gatekeeper of CCI.

To validate the robustness of these results, we repeated the analysis of CCI after downsampling the original data to 80% of the sample sizes for each condition group. We observed that the CCI networks and network centrality conclusions of MIF pathways are robust to downsampling (Figure 2, Supplementary Figure S2).

### 3.3 Cell type diversity of CCIs in the MIF pathway

We applied three cell type diversity statistics of CCIs, including degree, diversity, and entropy, to study the diversity in interactions of each cell type across five groups in COVID-19.

**FIGURE 3:**
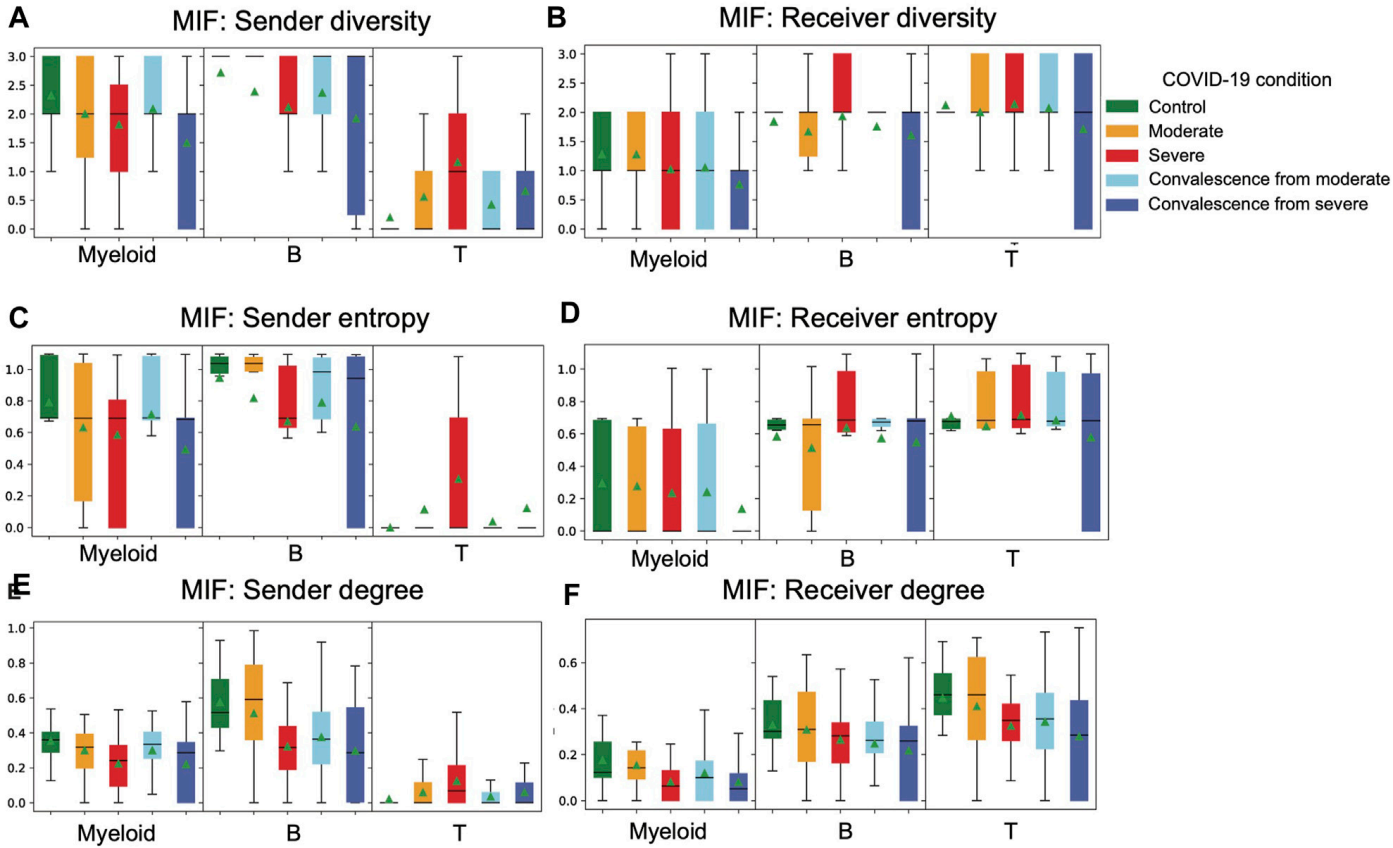
Distribution of cell type diversity statistics in different COVID-19 conditions are visualized. **(A)** Distribution of sender diversity in MIF pathway. There is a consistent pattern of decreasing diversity from control to moderate to severe groups and from moderate convalescent to severe convalescent groups in myeloid and B cell types as senders. The pattern in T cell type is different from the patterns in myeloid and B cell types. **(B)** Distribution of receiver diversity in the MIF pathway. There is a decreasing pattern of diversity from control to moderate to severe to moderate convalescence and to severe convalescence groups in myeloid as a receiver. **(C)** Distribution of sender entropy in the MIF pathway. The patterns are similar to sender diversity. **(D)** Distribution of receiver entropy in the MIF pathway. The patterns are similar to receiver diversity. **(E)** Distribution of sender degree in the MIF pathway. The patterns are similar to sender diversity and sender entropy. **(F)** Distribution of receiver degree in the MIF pathway. When B cells are receiver cells, there is a consistent decreasing trend of receiver degree from control all the way to severe convalescence groups.

We first calculated the cell type diversity to measure the number of different cell types with which sender or receiver interacts (Figures 3C,D). A higher cell type diversity means the cell type interacts with more cell types, indicating a higher variability of interactions. We observed that T cells are the cell type with the highest mean cell type diversity as receivers but the lowest cell type diversity as senders. When comparing between groups of conditions, when myeloid cells or B cells are senders, we found a clear decrease in mean diversity from control to moderate to severe groups. Also, when these two cell types are senders, mean diversity decreases from the moderate convalescence group to the severe convalescence group. These findings indicate that as disease progresses, myeloid and B cells as senders tend to interact with fewer receivers. When T cells are sender cells, there is an increase in diversity from control to moderate to severe groups and an increase from the moderate convalescence group to the severe convalescence group. This means that unlike myeloid and B cells, T cells tend to interact with more cell types in severe patients. Interestingly, when T cells are sender cells, we observed that the mean of cell type diversity in moderate and severe convalescence groups are less than moderate and severe groups respectively, which indicates a loss of interaction diversity in convalescent stages. Looking at diversity of receiver cell types, we observed a decreasing pattern of diversity of myeloid as receiver cells from control and moderate groups to severe groups, and the diversity in two convalescence groups are less than their corresponding groups in disease. This indicates that receiver diversity decreased in the severe group and the convalescence groups. We also observed that when B or T cells are receiver cell types, the variation of diversity is the highest in the convalescence from severe group.

We then calculated the cell type entropy to measure whether a sender interacts evenly with different receivers in strengths (Figures 3E,F). A high cell type entropy means the cell type interacts evenly with different cell types, while a lower entropy means the interactions of a sender are concentrated on few receivers. Comparing the cell type entropy across different COVID-19 conditions, we observed that both diversity and entropy had a similar trend. For example, the decreases in diversity in myeloid and B cells as senders from control to moderate to severe also occur in entropy. This means that a sender (receiver) in a typical sample will not have extremely uneven interactions with its receivers (senders). Otherwise, if myeloid cells are communicating with all three cell types but the interactions are concentrated towards T cells, then its entropy will be close to minimum but diversity will be three, which is maximum, causing the two measures to be significantly different.

As a standard measure of interaction strength, the in-degree and out-degree for all nodes (cell types) across all five condition groups are also calculated. The in-degree of a node is the total amount of interactions a receptor receives from all senders. Similarly, out-degree is the total amount of interactions a sender sends to all receivers. Interestingly, the trends we observed in cell type diversity and entropy were similar in senders but not so in receivers. When T cells are receiver cells, we observed a decrease from control to moderate to severe groups and from moderate convalescence to severe convalescence groups. But the consistent decreasing pattern from control to moderate to severe groups does not occur in receiver diversity nor receiver entropy of T cells. When B cells are receiver cells, there is a consistent decrease from control all the way to severe convalescence groups, which does not occur in the previous measures either. The aforementioned analysis implies that the three cell type diversity statistics can be used together to reveal a comprehensive picture of its interactions from three angles: interaction strength, number of senders or receivers the cell type interacts with, and the evenness of distribution of interaction with different senders or receivers. We applied these three cell type diversity metrics on a COVID-19 dataset to summarize the overall diversity trend of cell types in different disease conditions.

### 3.4 Node-to-node information flow in CCI networks in the MIF pathway

**FIGURE 4:**
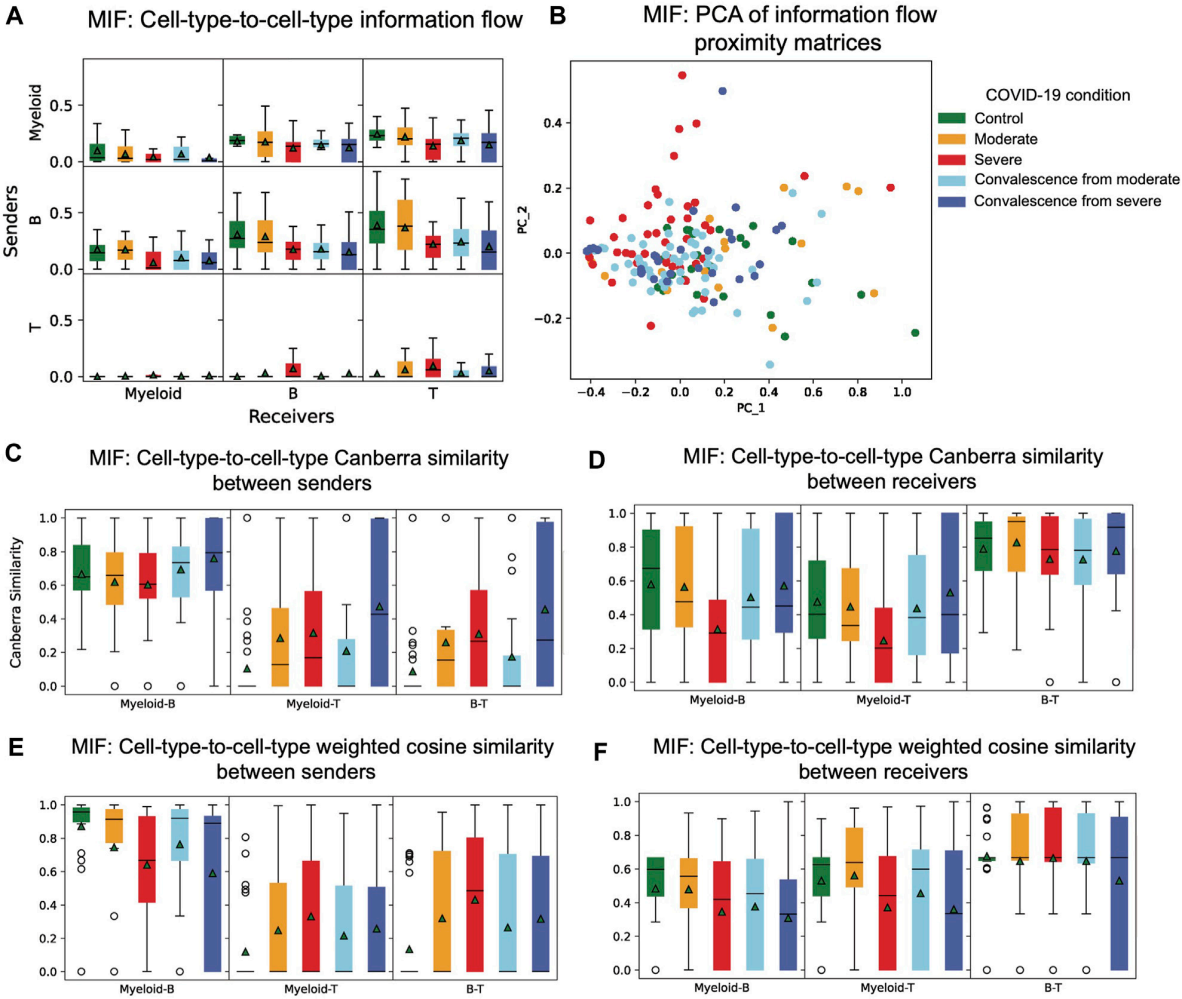
Distribution of cell-type-to-cell-type features. **(A)** Distribution of information flow between cell types of five COVID-19 conditions in the MIF pathway. When B cells are the sender cell type in each group of condition, mean and variance of information flow increases from myeloid to B cells to T cells as receiver cell types. **(B)** PCA of information flow proximity matrices of five COVID-19 conditions in the MIF pathway. There is a clear pattern that differentiates severe patients from healthy controls and moderate patients. **(C)** Distribution of cell-type-to-cell-type modified Canberra similarity between sender cell type pairs of five COVID-19 conditions in the MIF pathway. Myeloid and B cell types are similar as senders. **(D)** Distribution of cell-type-to-cell-type modified Canberra similarity between receiver cell type pairs of five COVID-19 conditions in the MIF pathway. B and T cell types are similar as receivers. **(E)** Distribution of cell-type-to-cell-type weighted cosine similarity between sender cell type pairs of five COVID-19 conditions in the MIF pathway. When comparing myeloid and B as sender pairs, there is a decreasing pattern from control to moderate to severe and from moderate convalescence to severe convalescence groups. On the contrary, when comparing myeloid and T cells or B cells and T cells sender pairs, the patterns are the opposite. **(F)** Distribution of cell-type-to-cell-type weighted cosine similarity between receiver cell type pairs of five COVID-19 conditions in the MIF pathway. When comparing myeloid and B as receiver pairs, there is a decreasing pattern from control and moderate groups to severe and to moderate convalescence and to severe convalescence groups.

To study the total amount of information flow among different nodes in CCI graphs, we calculated the information flow proximity matrix of each patient in the COVID-19 dataset. Comparing the distributions of node-to-node or cell-type-to-cell-type information flow across the five groups of different disease conditions, we observed several patterns (Figure 4A). When myeloid cells are sender cells, there is a decrease in mean information flow from control to moderate to severe groups and from moderate convalescence to severe convalescence groups. This observation indicates that the importance of myeloid cells as sender cells in a network decreases in these groups. The mean information flow from B cells to each cell type is lower in severe patients than in healthy and moderate COVID-19 patients and is lower in severe convalescence patients than in moderate convalescence patients. We also made an interesting observation that when B is the sender cell type, in each of five groups of conditions, mean and variance of information flow increases from myeloid to B cells and from myeloid cells to T cells as receivers. We found that T cells mainly function as receivers rather than senders, and there is almost no information sending from T cells to myeloid in all five groups.

A principal component analysis (PCA) is then performed on the information flow proximity matrices of each patient. Patients are then visualized with respect to the first two principal components (Figure 4B). While nonlinear projection methods, such as t-distributed stochastic neighbor embedding (tSNE) or UMAP can be used, we used linear PCA for visualization to maximize interpretability, as tSNE and UMAP require careful calibration of hyperparameters to ensure robust results (Supplementary Figure S3). We can see that with respect to information flow, severe patients differentiate from healthy controls and moderate patients.

### 3.5 Modified Canberra node-to-node similarity in CCI networks *via* the MIF pathway

To study how similar two cell types as senders (receivers) are with respect to their interaction strength with each receiver (sender), we calculated the modified Canberra proximity matrix for each patient and plotted the distribution of modified Canberra similarity of each pair of senders or receivers (Figures 4C,D). There are several interesting observations we can make. First, myeloid cells and B cells as senders have the highest similarity among sender pairs in all five groups. Second, myeloid cells and T cells as senders have a similar pattern with B cells and T cells as senders across different groups. These two findings may indicate that for each receiver, myeloid cells and B cells are very similar as senders, but T cells seem to function differently as senders. We also observed that of all possible receiver pairs, B cells and T cells have the highest similarity. Myeloid cells and B cells or myeloid cells and T cells as receivers have similar patterns of similarity across different groups. Similarly, this indicates that for each sender, B cells and T cells as receivers act similarly, but myeloid cells may function differently. We then compared the differences in five different groups of conditions. When comparing myeloid cells and T cells as senders, or B cells and T cells as senders, there is an increase of mean modified Canberra similarity from control to moderate to severe groups and from moderate convalescence to severe convalescence. This indicates that as disease progresses, the relative difference of interactions of myeloid cells and T cells as senders tend to decrease as a response to disease. Similarly for B cells and T cell types, when observing patterns in receiver pairs, we found that severe group has the lowest similarity between myeloid cells and B cells and between myeloid cells and T cells. Thus in severe patients, the relative difference of interactions between myeloid cells and B cells or between myeloid cells and T cells as receivers decreases.

### 3.6 Weighted cosine node-to-node similarity in CCI networks in the MIF pathway

The weighted cosine similarity between two senders measures if the senders have similar relative composition of ligands. A high similarity between two senders indicates that interactions of both senders with the same receiver are highly positively correlated. We also have a similar interpretation for weighted cosine similarity of two receivers. We calculated weighted cosine proximity matrices of each patient in the dataset and plotted the distribution of similarity of each pair of senders and receivers (Figures 4E,F). In comparing myeloid and B as both senders and receivers, we observed a decrease in mean similarity from control to moderate to severe groups and from moderate convalescent to severe convalescent groups. This means that in the progression of disease, the relative composition of ligand genes in myeloid cells and B cells start to differ. On the contrary, when comparing myeloid cells and T cells or B cells and T cells as senders, we found an increase from control to moderate to severe groups and from moderate convalescent to severe convalescent groups. This suggests that as disease progresses, myeloid cells and T cells and B cells and T cells start to recruit similar receivers. These trends also indicates that while the functions of myeloid and B cells as senders alters across COVID-19 progression, their function approaches that of sender T cells.

We also observed a significant decrease in of mean weighted cosine similarity of myeloid cells and T cells as receivers in severe patient group, compared to control and moderate groups. Thus, as disease progresses, the relative composition of receptor genes changes dramatically in myeloid cells and T cells. Overall, we found that myeloid cells and B cells as senders and B cells and T cells as receivers are still the most similar pairs of senders and receivers under this measure, as they have the highest mean weighted cosine similarity in all the five groups.

### 3.7 Diversity of CCI networks in the MIF pathway

Following Section 2.3.3, we used the following distances to characterize distance between CCI networks:

1) Apply root Euclidean distance between information flow proximity matrices;
2) Apply root Euclidean distance to sender and receiver node-to-node modified Canberra proximity matrices and take $d=\sqrt{d_{\mathrm{sender}}^{2}+d_{\mathrm{receiver}}^{2}}$ as the combined distance.
3) Apply root Euclidean distance to sender and receiver weighted cosine proximity matrices and use $d=\sqrt{d_{\mathrm{sender}}^{2}+d_{\mathrm{receiver}}^{2}}$ as the combined distance.
4) Modified Canberra distance between CCI adjacency matrices.

The measures (1), (2), and (3) measure the root Euclidean distances between node-to-node affinity matrices, thus quantifying the overall cell-type-to-cell-type differences.

**FIGURE 5:**
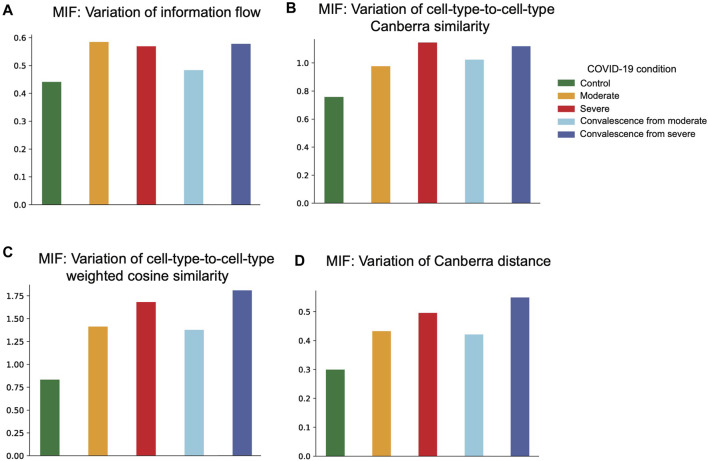
Network variability based on four distance measures. **(A)** Network variability based on information flow distance. The variability in moderate and severe groups is higher than that in the control group. The variability in the severe convalescence group is higher than that in the moderate convalescence group. **(B)** Network variability based on cell-type-to-cell-type modified Canberra distance. There is an increasing pattern of variability from control to moderate to severe groups and from moderate convalescence to the severe convalescence group. **(C)** Network variability based on cell-type-to-cell-type weighted cosine similarity. There is an increasing pattern of variability from control to moderate to severe groups and from moderate convalescence to the severe convalescence group. **(D)** Network variability based on graph-to-graph modified Canberra distance. There is an increasing pattern of variability from control to moderate to severe groups and from moderate convalescence to the severe convalescence group.

Then we calculated variations of network measures within each health condition group (Figure 5). In the case of information exchange, we can interpret a higher variation within a condition as a larger difference in network level node-to-node importance. We observed a consistent increase in network diversity from healthy controls to severe patients. These findings indicate that CCI patterns in COVID-19 patients are more diverse compared with healthy controls. Under the aforementioned measures, we also observed a higher variation in the convalescent group from severe symptoms than that in the moderate convalescent group.

## 4 Discussion

As more large-cohort scRNA-seq studies, both in terms of the number of cells and the number of tissue samples, become available, it is important to account for and analyze the individual-sample heterogeneity, both within and across the relevant biological groups, such as disease status. To this end, we derived different diversity measures of CCI and used them to study the diversity of CCI in a scRNA-seq dataset of COVID-19 that was sampled from 181 patients across five conditions, focusing in particular on the MIF pathway. By representing CCC networks as networks, where cell types are nodes and interaction scores are directed, weighted edges, we studied three categories of network features: single node (cell type), node-to-node level, and network-level. We then studied variations across networks in different groups of conditions.

At the node level, we analyzed both sender and receiver variation and entropy. The sender (receiver) variation measures the number of receivers with which (sender) a sender (receiver) cell type interacts, while sender (receiver) entropy measures the uniformity of outgoing (incoming) interaction strengths with different receivers (senders). Together with sender (receiver) degrees, these three diversity measures provide a comprehensive picture of diversity at the cell type (node) level.

Next, we studied pairwise node-to-node or cell-type-to-cell-type level features, which measures either amount of total information exchange from one type to another or the similarity of two senders (receivers). The first measure, information flow from cell type *i* to *j*, measures the total amount of information sending from cell type *i* that is received by *j*. The second measure, modified Canberra node-to-node similarity, measures the relative difference of interactions between two senders (receivers). The third measure, weighted cosine node-to-node, measures if the senders (receivers) have similar relative composition of ligands (receptors). The modified Canberra and weighted cosine node-to-node similarities can serve as complementary measures of node-to-node similarities. The former provides similarity in relative interaction strengths, and the latter provides similarity in relative composition of relevant ligands or receptors.

Finally, using different metrics between CCI graphs, we studied total variation across graphs in different patient groups comprising conditions. We found that there was a consistent pattern in an increase of variation in COVID-19 groups than in control groups, suggesting a more diverse CCI reaction pattern in diseased patients. The aforementioned analysis of CCI networks from three angles, namely, single node, node-to-node, and network levels, can be applied to analyze any other pathways in COVID-19. These proposed methods on analyzing CCI diversities within different groups of conditions could also be applied to sub cell types to reveal biologically meaningful results, with the caveat that introducing more cell types can increase the amount of noise from CCI output (Supplementary Figure S4-S8). We noted that using different CCI tools, such as CellCall (Zhang et al., 2021), may result in different CCI output for individual patients, and the overall trend of patient-to-patient variability in CCI will remain, emphasizing the importance of methodologies and analyses that measure the diversity of CCI across cohorts of networks.

One technical limitation of the diversity measures proposed for CCI is that the majority of networks identified signaling pathways were sparse, indicating very few interactions. As cell types were more isolated for these pathways, the node-to-node analyses fail to recognize meaningful patterns. For example, the cosine similarity between two sender cell types is almost exclusively 0 and 1, and the majority of interactions do not overlap. This phenomenon pertains even with weighted cosine similarity for very sparse networks. As a result, the diversity of sparse networks based on these measures cannot be interpreted meaningfully, in the way that dense networks can be. One can use hand-selected features in sparse networks to condense the interaction information, but this may result in loss of biological interpretation after feature selection. In this direction, future work will be needed to address this limitation of sparsity when studying sample-to-sample graph diversity, while retaining biological interpretability of these methods.

## Data Availability

Publicly available datasets were analyzed in this study. This data can be found at : data are available at the NCBI GEO database with accession number GSE158055 and can be accessed at the following link: https://www.ncbi.nlm.nih.gov/geo/query/acc.cgi?acc = GSE158055.
