# Supplementary material for: Analyzing network diversity of cell–cell interactions in COVID-19 using single-cell transcriptomics
Source: Front Genet. 2022 Aug 29;13:948508. doi: 10.3389/fgene.2022.948508 (PMC9465179; doi:10.3389/fgene.2022.948508)
Supplement: Supplementary file 1 [file DataSheet1.PDF]

Supplementary Material

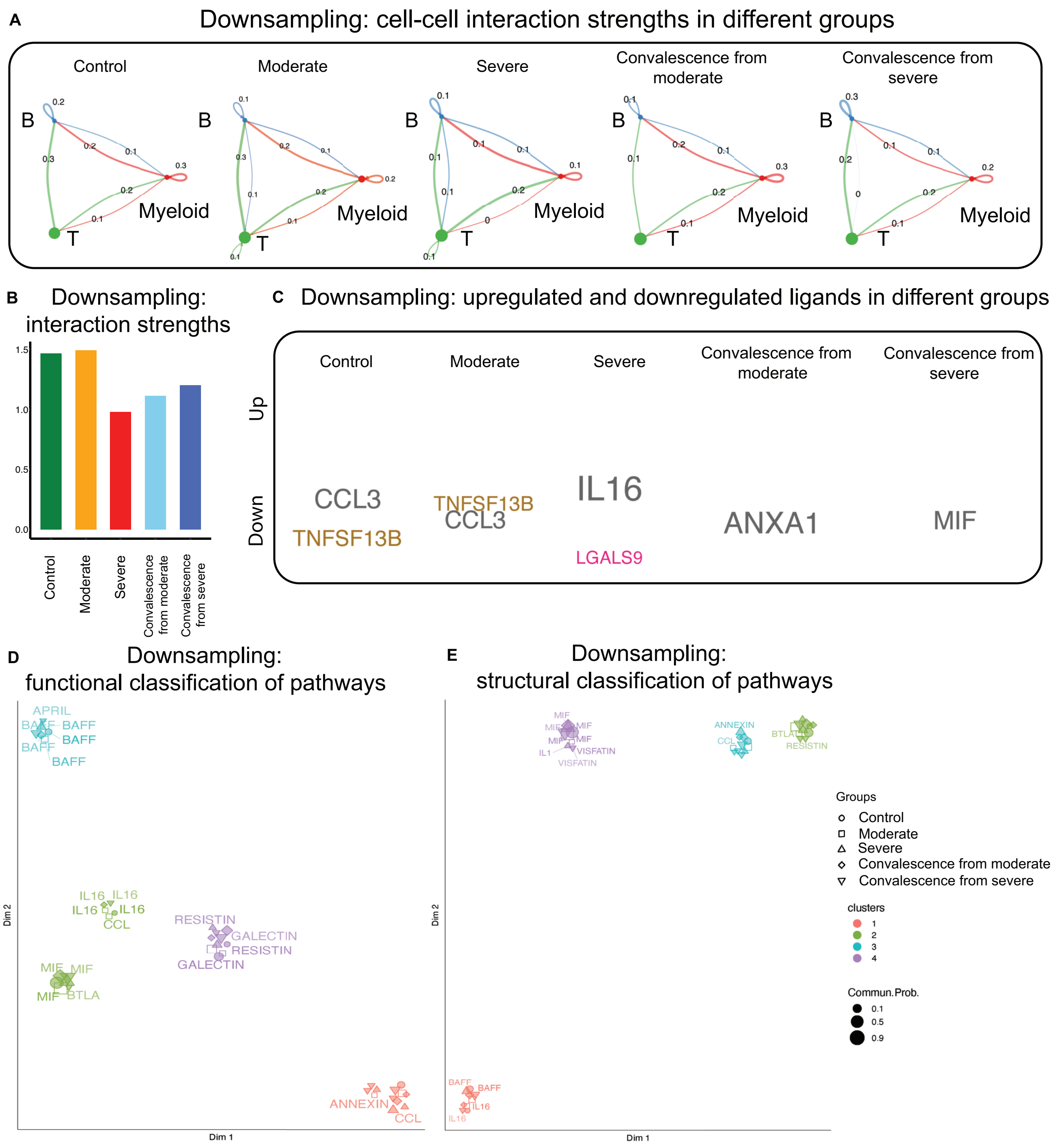

Figure S1. CellChat analysis of all pathways after downsampling. A. CCI interaction strengths of each downsampled condition, as obtained by random sampling of 80% of each condition group. Each directed edge represents an interaction and its colour corresponds to the color of the sender cell type. **B**. Total interaction strength in different downsampled groups. The total interaction strengths from the downsampled Control and Moderate groups are higher. **C**. Upregulated and downregulated ligands for each downsampled condition group **D**. Projection of signaling pathways onto a two-dimensional manifold according to their functional similarity. Each dot represents the interaction network of one signaling pathway. Different colors represent different inferred groups of signaling pathways. **E**. Projection of signaling pathways onto a two-dimensional manifold according to their structural similarity. Each dot represents the interaction network of one signaling pathway. Different colors represent different inferred groups of signaling pathways.

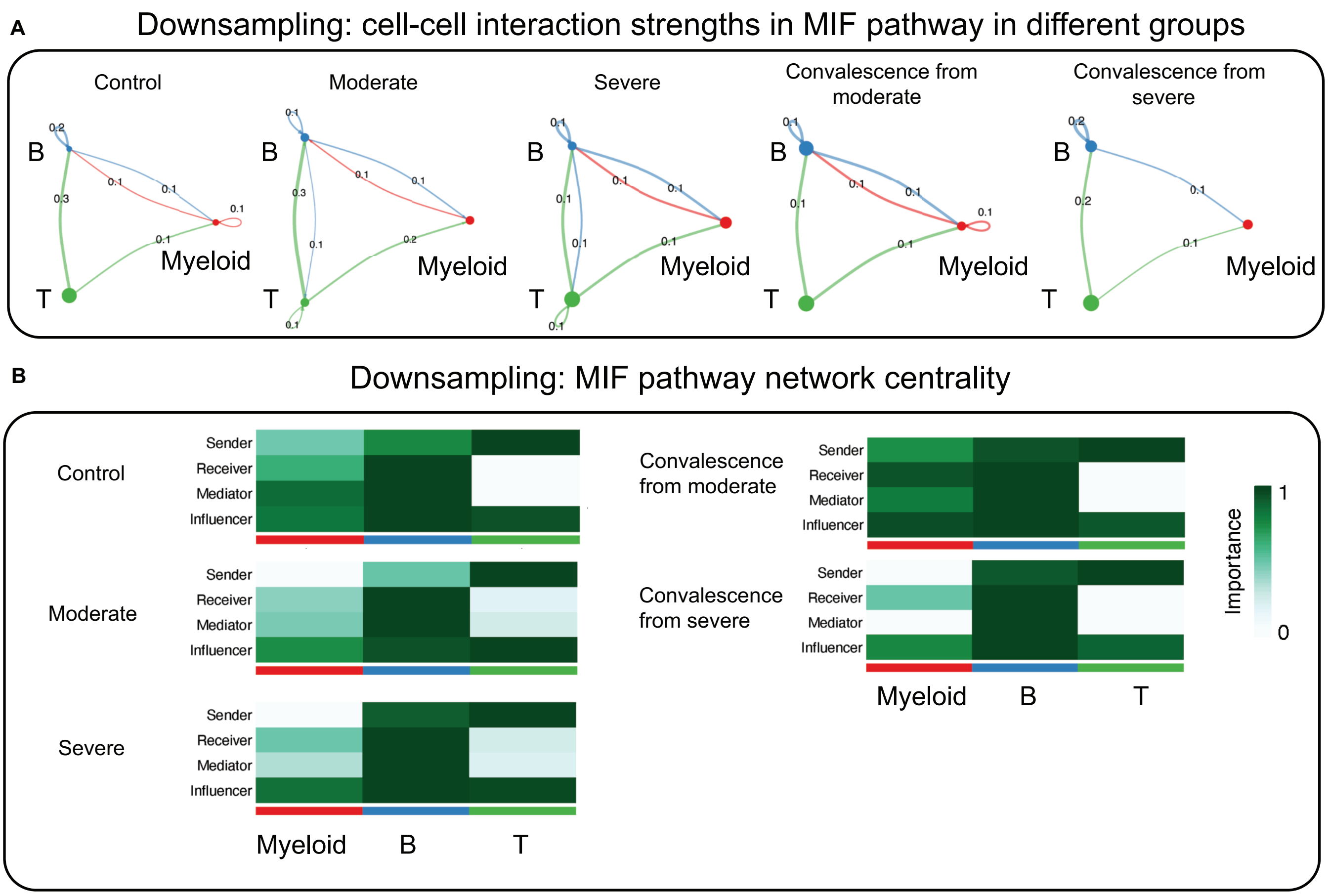

Figure S2. CellChat analysis of MIF pathway after downsampling. A. CCI strengths of each downsampled condition group. Each edge represents an interaction, where the color of the edge matches the sender cell type. **B**. Network centrality scores of downsampled condition groups in MIF pathway. In downsampled severe and convalescence from severe groups, Myeloid cells are not implied to be mediators.

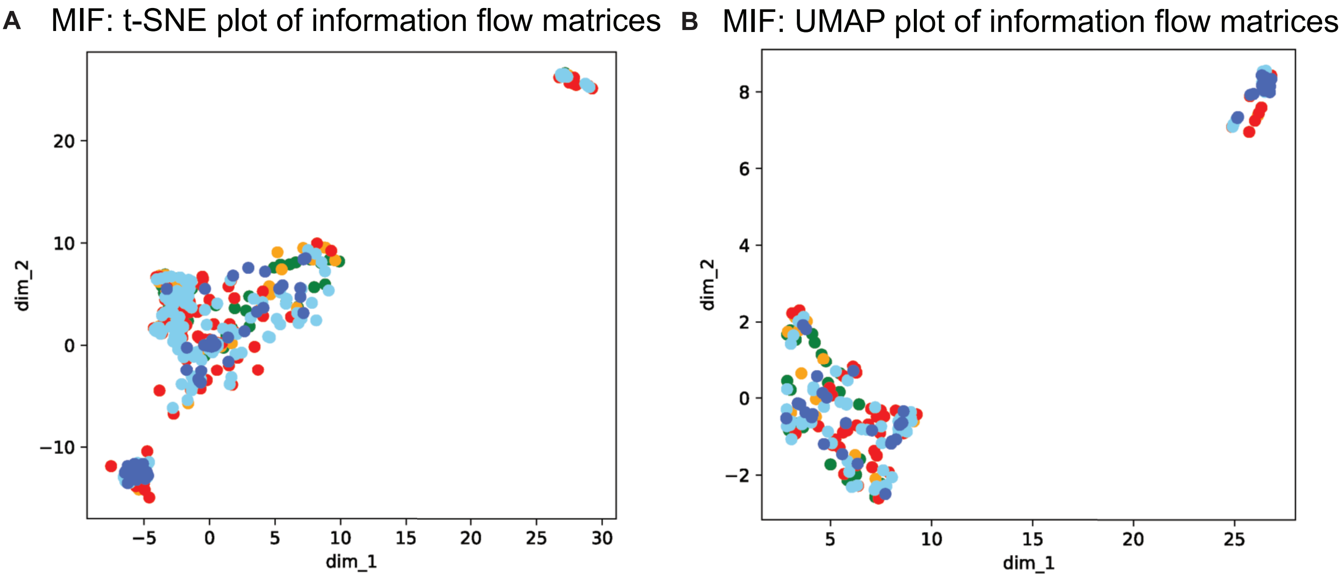

Figure S3. Visualization of information flow using nonlinear embeddings. A. t-SNE plot of information flow matrices of three cell types (Myeloid, B, T) in MIF pathway. **B**. UMAP plot of information flow matrices of three cell types (Myeloid, B, T) in MIF pathway.

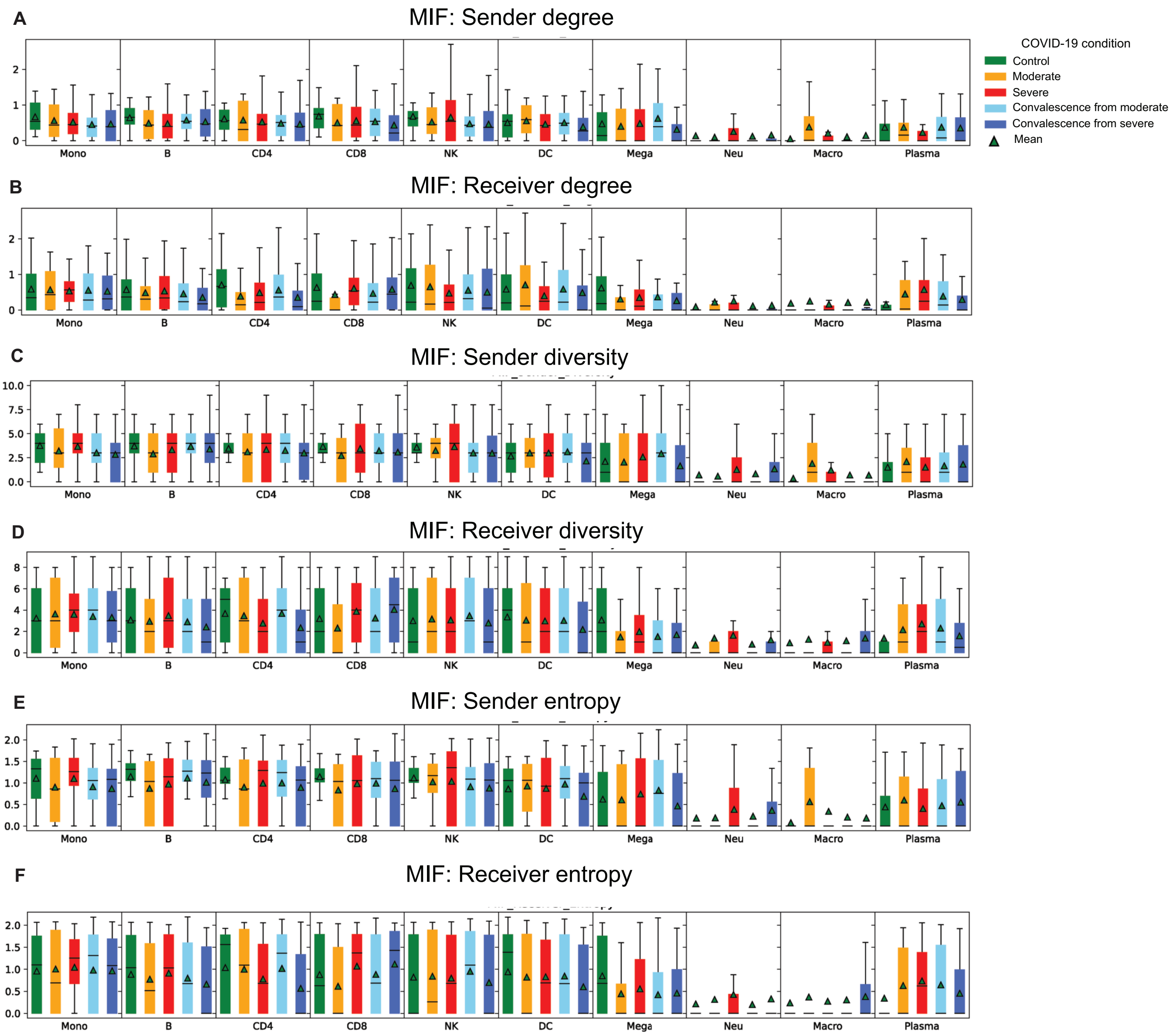

Figure S4. Distribution of ten cell types diversity statistics in different COVID-19 conditions are visualized. A. Distribution of sender degree in MIF pathway. **B**. Distribution of receiver degree in MIF pathway. **C**. Distribution of sender diversity in MIF pathway. **D**. Distribution of receiver diversity in MIF pathway. The patterns are similar to receiver diversity. **E**. Distribution of sender entropy in MIF pathway. **F**. Distribution of receiver entropy in MIF pathway.

A

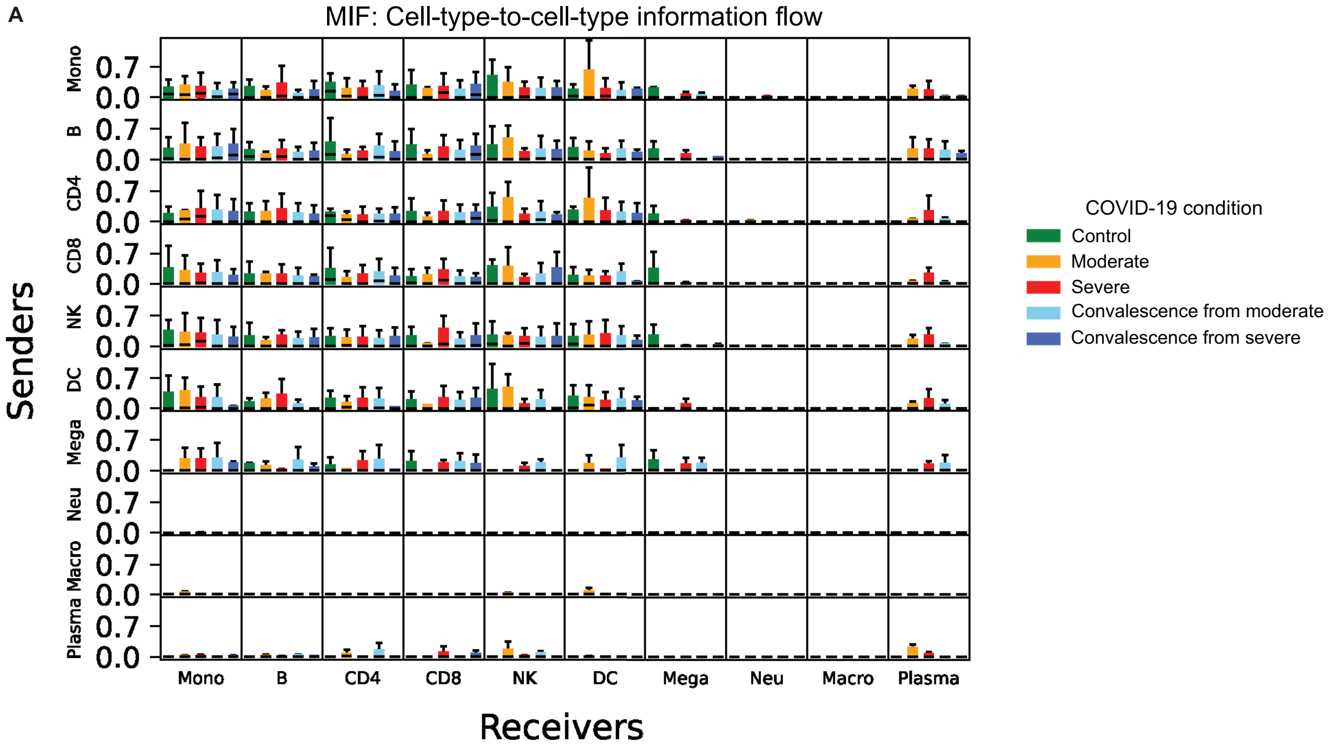

Figure S5. Distribution of node-to-node information flow statistic of ten cell types in MIF pathway in different COVID-19 conditions and comparison of nonlinear projection of information flow proximity matrices of three cell types. A. Distribution of node-to-node information flow in MIF pathway in five condition groups.

A

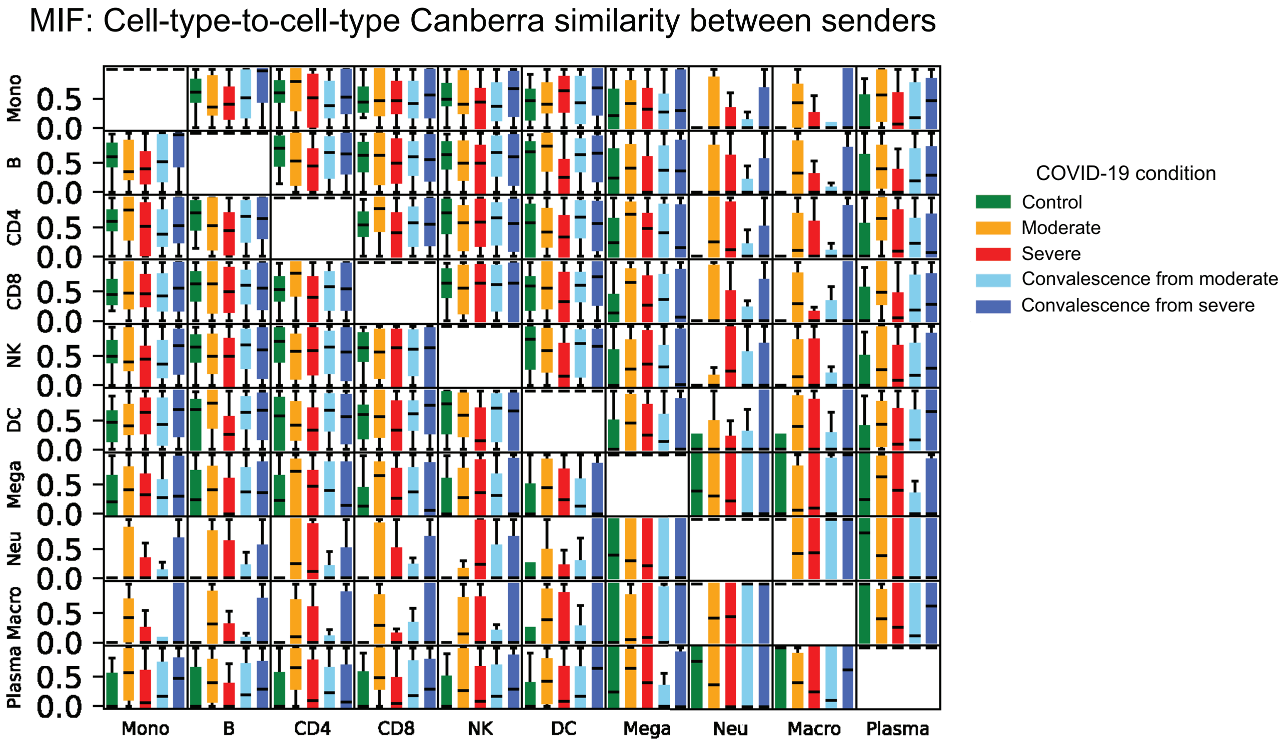

B

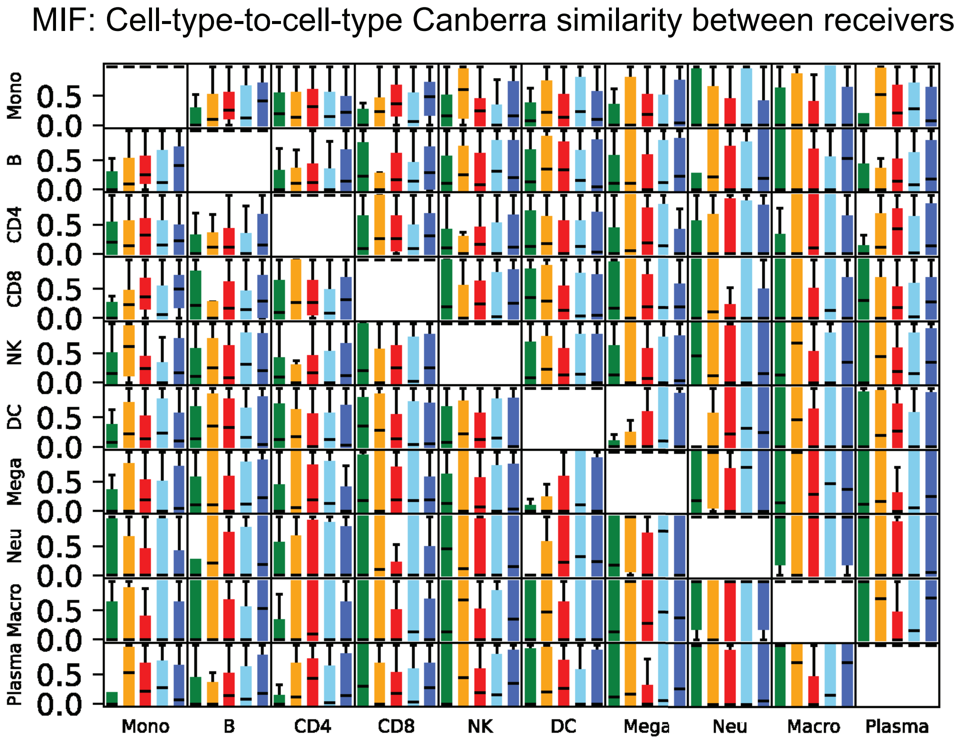

Figure S6. Distribution of node-to-node Canberra similarity of ten cell types in different COVID-19 conditions are visualized. A. Distribution of node-to-node Canberra similarity between senders in five condition groups. **B**. Distribution of node-to-node Canberra similarity between receivers in five condition groups.

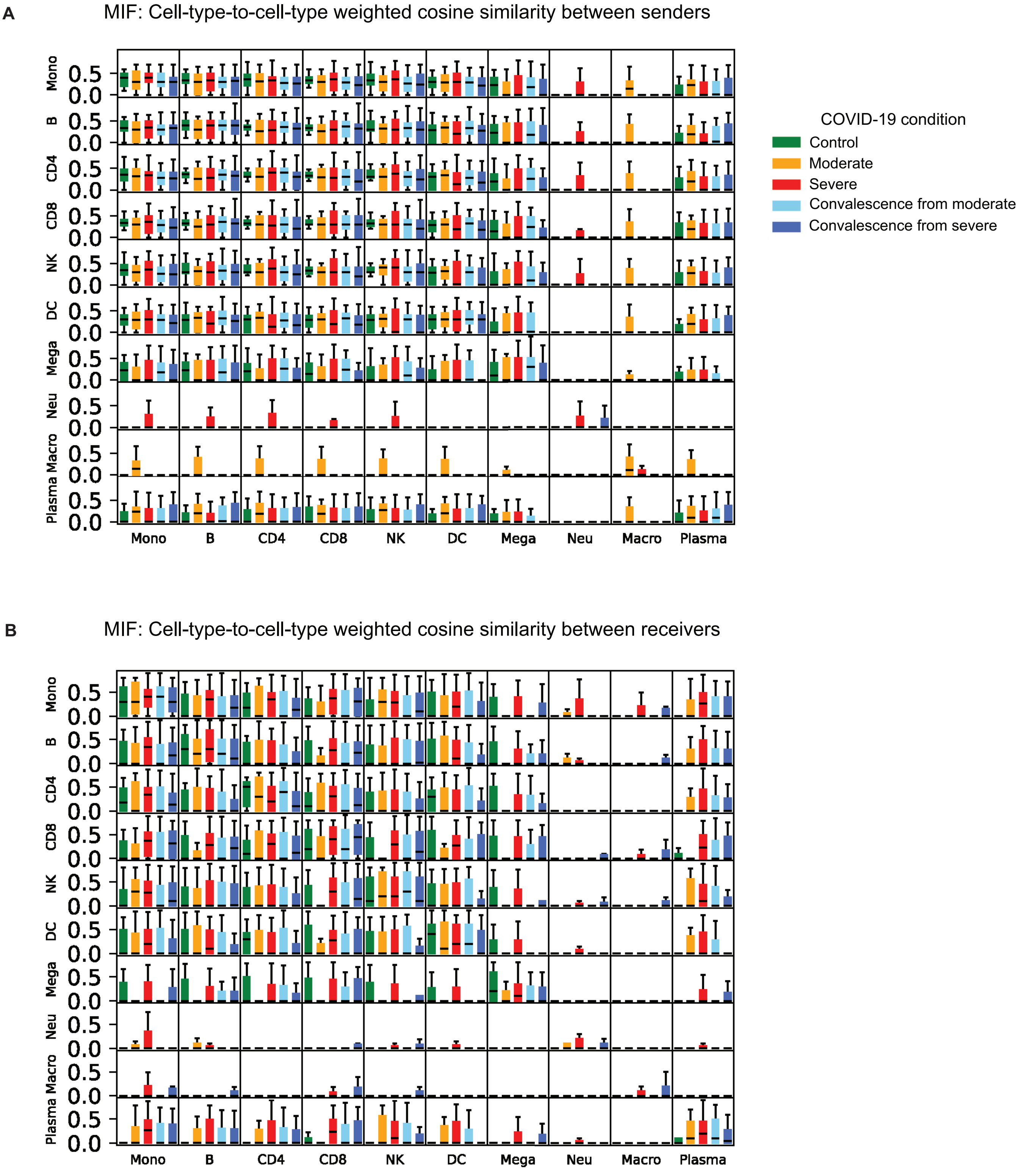

Figure S7. Distribution of node-to-node weighted cosine similarity of ten cell types in different COVID-19 conditions are visualized. A. Distribution of node-to-node weighted cosine similarity between senders in five condition groups. **B**. Distribution of node-to-node weighted cosine similarity between receivers in five condition groups.

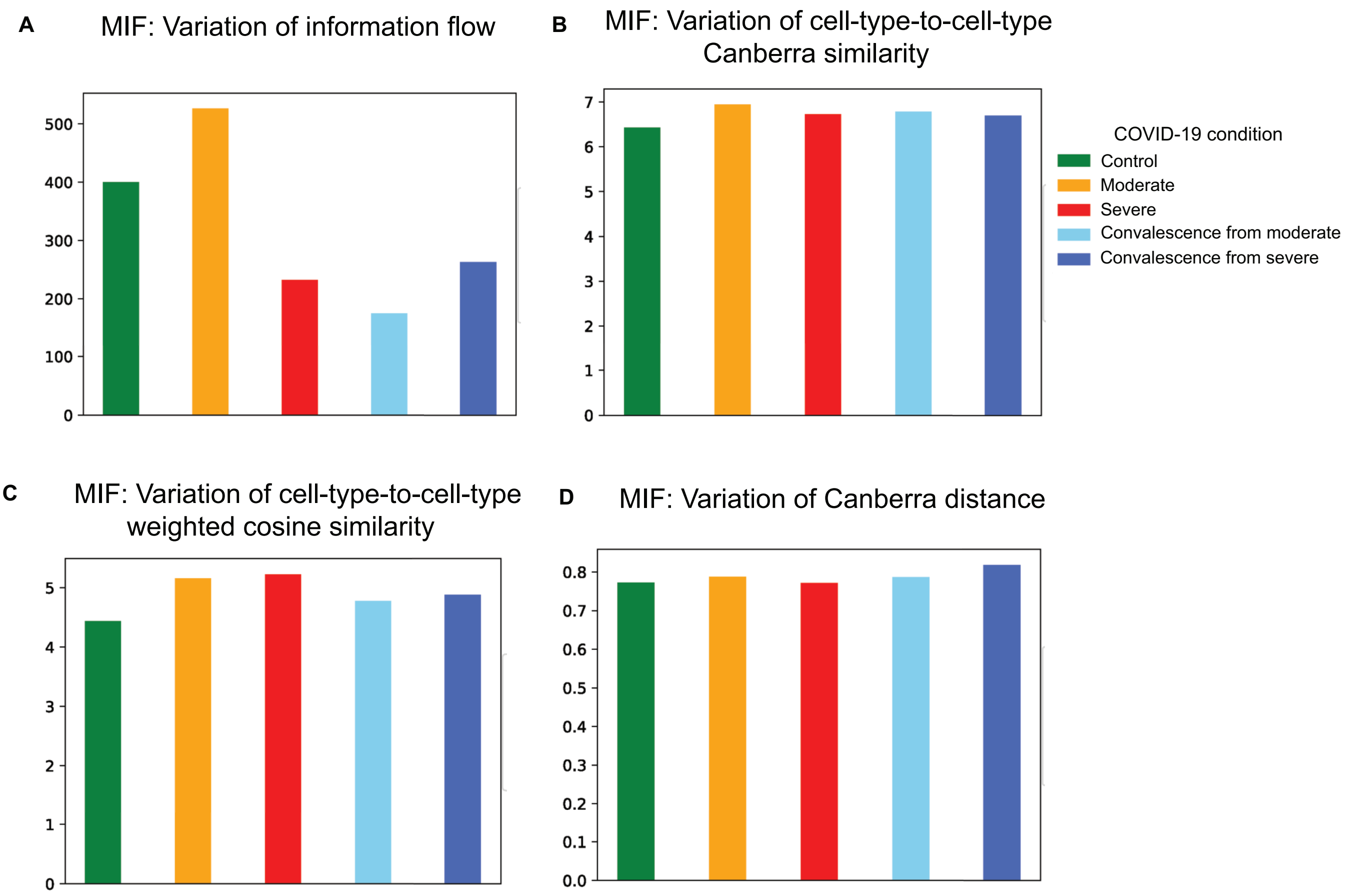

Figure S8. Network variability based on four distance measures in ten cell type networks. A. Network variability based on information flow distance. **B**. Network variability based on cell-type-to-cell-type Canberra distance. **C**. Network variability based on cell-type-to-cell-type weighted cosine similarity. **D**. Network variability based on graph-to-graph Canberra distance.
